# Let’s Talk About it: The Utility of Formalized Support for Medical Residents

**DOI:** 10.34172/ijhpm.2023.7463

**Published:** 2023-08-13

**Authors:** Rachel Gifford, Tiuri R. van Rossum, Bram Fleuren, Daan Westra

**Affiliations:** ^1^Department of Health Services Research, Care and Public Health Research Institute (CAPHRI), Faculty of Health, Medicine and Life Sciences, Maastricht University, Maastricht, The Netherlands; ^2^School of Health Professions Education, Faculty of Health, Medicine and Life Sciences, Maastricht University, Maastricht, The Netherlands; ^3^Department of Work and Social Psychology, Faculty of Psychology and Neuroscience, Maastricht University, Maastricht, The Netherlands

**Keywords:** Hospital Management, Clinician Burnout, Well-Being Support, The Netherlands, Resident Education

## Abstract

Medical residents are significantly impacted by burnout and depression. Recent events have only further increased the pressure and demands on the healthcare sector, intensifying the burden facing residents and posing a threat to residents’ well-being. As a result, significant efforts are being made to provide formalized support and well-being programs. Yet, emergent evidence indicates that residents do not sufficiently utilize this form of support. Considering the organizational investment and focus on formalized support programs, we conducted a mixed-method study to investigate residents’ utilization of formalized well-being support, and potential reasons for non-use. Our study was conducted during a period of increased work burden and stress for medical residents, where formalized support was specifically offered and targeted to medical staff. Our findings confirm earlier results of low support utilization and point to the importance of informal support mechanisms, in particular peer support. We conclude by discussing the role of managers and educational programs in facilitating a positive cultural shift to promote and support residents in seeking support.

## Background

 Medical residents are profoundly affected by burnout and depression. Depending on the definition, measurement and specialty, the estimated prevalence of depression and burnout for residents ranges from around 30%^1^ to 50%, respectively.^2,3^ These conditions that undermine well-being have serious implications for resident productivity,^4^ motivation and performance,^5^ as well as patient safety.^6^ Self-care and support seeking behavior of residents are thus important for patient outcomes, quality of care,^7^ and for residents themselves.^8^ The importance of resident well-being is reflected in the increase of attention in scholarly articles and the urgency in which national and local residency programs are implementing well-being interventions.^9^ However, while some of these interventions have positive results, there are also signs that residents may not sufficiently utilize formal support.^10^

 Recent events and reports of intensifying work pressures puts a magnifying glass on the issue of resident well-being and support. For example, the recent COVID-19 crisis threatened residents’ well-being due to increased workload and significant challenges in their training, education, and responsibilities.^11^ In recognition of the burden and intensity of work that residents and other healthcare workers face, significant efforts are being made to provide well-being and formalized support programs for medical staff.^12^ As Lai et al,^13^ indicate, a sense of support can have a positive impacted on residents’ well-being. However, Zoorob et al,^10^ show that over 40% of surveyed residents who were offered formal support during the COVID-19 period did not find it useful or make use of it. However, the reasoning behind low support utilization remains unclear. This prompts us to consider the utility of formal support and if residents experience barriers to its use. Examining the utility of formalized support can lead to valuable insights for the way we give attention to well-being in a setting that is constrained by the balance between education and clinical care.

## Methods

 In order to set the basis for future research, we conducted a small scale mixed-method study to identify whether residents indeed do not utilize formal psychological support and what potential underlying reasons might be for non-use. The data utilized for this study is part of a larger mixed-method study of how hospitals in a heavily hit region of the Netherlands adapted to and coped with the COVID-19 crisis that has been ongoing since September 2020.^14^ To ascertain support use, we conducted analysis of a survey which included a subsample of medical residents, and assessed the utilization of formalized support programs across five hospitals in the Netherlands.^14^ To examine potential antecedents of non-use, the first author conducted nine interviews with residents, and medical specialists between September 2020 and April 2021. More specifically, we conducted an interpretive analysis, reviewing responses of (five) medical residents and (four) medical specialists to questions that focused on impact of the crisis, available support, use of support and support mechanisms, well-being in general. We utilize emergent insights regarding the well-being and support seeking behaviors of residents to support our current theorizing and to call for future research into this issue.

## Results

###  Uptake of Formal Support

 From the interviews, medical specialists suggested that residents had taken on an enormous burden throughout the course of the crisis. Some participants emphasized the reality that residents were confronted with high emotional and mental burdens during frontline work, and in particular with patient death. The intensity of the work, the illness of the patients, and unfamiliar roles, wards, and tasks created additional burdens. However, while formal support was available for the interviewed participants (eg, via an internal psychosocial support team, support helplines, external professional psychologists on standby), the present data suggests that use of these interventions may have been low for this group,

 “*Yes, we always had, yes, one employee who actually came by every day to the A&E department: “is there someone who needs help?” or someone who just wanted to talk. But I have to say, I don’t know, I didn’t take advantage of it. I don’t think much of our group. But I think it was more for the other specialties” *[Resident].

 Quantitative questionnaire data suggested that in the subsample of 24 residents, only 2 indicated that they used formal support during the first COVID-19 peak in the Netherlands (19 indicated they did not, 3 did not reply). Of the 19 residents who indicated that they did not make use of formal support, none even considered using it. Responses to other items related to support indicated that the residents in this subsample did receive informal support, mostly from their families, coworkers, supervisors, and in some cases dedicated professionals (eg, psychologists). As such, it seems using formal support is not common among residents.

 In addition, the residents in our qualitative sample downplayed the toll of the pandemic on themselves while still highlighting its negative reality for their colleagues, pointing to clear patterns in other groups who are not used to such intense work and patient death. Medical specialists on the other hand noted the impact of the pandemic on residents.

 “*We still need our residents because they do most of the work. But on the other hand, we need to let them breathe because they they can’t keep up anymore. So we changed the shifts completely and they now have, for example, four residents do the same work they used to do in the first wave with two residents, so we try to lower the impact and the stress levels. But still, it’s not enough. And we we are struggling with how to support them and make them feel safe” *[Medical Specialist, Internist].

 In particular, some specialists pointed to recognition of signs of burnout and potentially long-term psychological tolls in the intensive care unit group. However, it was sometimes difficult to figure out how to best support them.

###  Institutional Barriers to Formal Support 

 Low utilization of support was attributed by one medical specialist as being related to the interdependency between residents and supervisors. Particularly for residents, their dependence upon their supervisors and the need to show they ‘can handle it’ may deter the utilization of support when support seeking is perceived as ‘weak.’

 “*…on one hand, I think it’s really good as a sign that you should talk about it and that the possibility is there. But on the other hand, I think a lot of the residents didn’t feel like sharing because they’re in this dependency of us as supervisors. And their whole residency depends on how they function as a doctor. So if you’re going to tell you’re struggling, it would be a symptom of a failing” *[Medical Specialist Internist].

 This need to be seen as ‘tough’ was echoed by another specialist who indicated the utility of residents being tough enough to handle the sometimes brutal reality of a residency program.

###  Peer Support and Modeling Behavior for Spontaneous Coping

 Despite the efforts to promote formal support utilization, for physicians the preferred mode of sharing experiences and seeking support was within the peer groups. Some participants pointed to self-care and seeking peer support as a form of best practice. In groups where senior staff made an effort to share experiences and start an open conversation, a psychologically safe environment may be created for residents to also engage in sharing experiences and seeking support in such groups.

 “*I think in the psychosocial support team they were asked to also help in providing with some emotional training [for residents] and to [help them] anticipate what type of situation they would come in to, how to handle this. And also how to help each other and talk about this if you needed. So there was attention also to this mental part of the workload…the head of the department of internal medicine was quite aware of this. So he also approached us in the beginning phase, please join, [so] he and I went to their daily meeting and he introduced us to the entire group, I think there was 60 people there, and said ‘ok these people can be of support and so forth and I think that it’s very important to talk about this if you want to.’ So I think giving this example of that this is normal to talk about how this is impacting your mental wellbeing, by the leaders in the department, that’s a very important thing. That was I think the strength here” *[Head of psychosocial support team].

 The modeling by leading physicians may be seen as a crucial factor in setting a norm of help seeking behaviors.

## Discussion

 Organizational support is a valuable asset for resident well-being, however as our data indicates, and has been noted in other emergent research, even during times of increased work pressures and burdens, residents exhibit low numbers of formal support utilization^10^ or may not perceive formalized interventions as beneficial for their well-being.^15^ Although the present sample is limited by a small sample size, our utilization of two sources of primary data across several hospitals allows us to bring forward important insights and provide a good basis for future research that incorporates a more robust sample. Our data suggest that informal peer-support may be utilized the most by residents, and thus may prove more effective than more formalized modalities. This aligns with other research that has shown peer support to be the most utilized and useful form of support for physicians.^16^ With resources being distributed towards formalized support programs, it is essential to consider whether these are the best suited, or most effective, types of support. In the following sections we consider ways forward, focusing on the importance of building peer support competences in medical training, and the role of management in building a culture of self-care and normalizing seeking support to better support the well-being of residents.

###  Ways Forward

####  Building Peer Support Competences

 In light of our finding that informal support may be more desired and utilized than formal programs, we suggest that it becomes increasingly important to learn peer-supporting behavior during medical training. That is, while compelling evidence exists that peer and informal social connections (eg, among residents) may be an essential factor in improving resident well-being,^17,18^ there is little formal training of peer support in the medical curriculum.^19^ As such, there is an urgent need to give more attention to skills needed for such peer-support in the medical curriculum. Training residents in competences such as active listening, peer-support and emotional intelligence may facilitate the development of a climate of support and well-being^19^ more effectively than formal well-being interventions.

####  The Role of Management 

 Confirming earlier notions that residents do not actively seek support when experience stress at work,^20^ our study also highlights the important role of leaders in fostering a support seeking and safe culture. Increasing demands and work stress on residents in recent years has put a magnifying glass on already existing challenges within the medical field, including the challenge of retention^21^ and alarmingly high rates of burnout and depression among clinicians.^1^ It is therefore essential that organizations and leaders take an active role to consider and promote the cultural changes that need to occur to protect residents and safeguard their well-being.^22^ Open discussion and peer support is necessary to allow physicians to recognize moral distress^21^ and can support general well-being. However, there are signs that it is difficult for residents to make time for recovery or seek support in an already busy schedule and stigma of support seeking or expressing mental health concerns often keeps residents suffering in silence, fearful of negative repercussions.^22^

 Supervisors, and managers can promote positive cultural change by modeling support seeking behavior themselves, normalizing self-care^19^ and by signaling its importance with structural changes. For example, organizations can work to structurally incorporate or mandate ‘downtime’ in residents’ schedules to offer informal opportunities for debriefing, peer discussion, and recovery activities. Management can also work with clinical supervisors and team leaders to design opportunities for group reflections and discussion, eg, biweekly or monthly. Such group reflections should be attended by, and ideally led, by senior staff to promote an open culture and reduce residents’ fears of sharing their own struggles.^19,22^ This can help build in necessary encouragement for health care providers to ‘talk about’ their own support needs so that these needs can be adequately addressed by the organization.

## Ethical issues

 Ethical approval was granted for this study by Maastricht University’s Research Ethics Committee (FHML-REC/2020/110).

## Competing interests

 Authors declare that they have no competing interests.

## Funding

 This work was supported by the Netherlands Organization for Health Research and Development (ZonMw) under grant number 1043002201001.
